# Risk Factors for Food Insecurity among Older Adults in India: Study Based on LASI, 2017–2018

**DOI:** 10.3390/nu15173794

**Published:** 2023-08-30

**Authors:** Joelle H. Fong

**Affiliations:** Lee Kuan Yew School of Public Policy, National University of Singapore, Singapore 259771, Singapore; j.fong@nus.edu.sg

**Keywords:** food insecure, nutrition, population aging, India, social isolation

## Abstract

Background: Food security is linked to the nutritional status and well-being of older adults. India is a rapidly aging nation that ranks highly on the 2022 Global Hunger Index. This paper examines the prevalence and risk factors of food insecurity in India’s older population. Methods: We used data from the 2017–2018 Longitudinal Aging Study in India. The sample size was 31,532 adults aged 60 years and above. Food insecurity was measured using a four-item version of the Food Insecurity Experience scale. Multivariable logistic regressions using individual-level weights were implemented to assess the risk factors of food insecurity. Results: The prevalence of food insecurity was 10.5% in the weighted sample. Sociodemographic factors were important in explaining food insecurity. Older adults who were male, younger, lowly educated, socially disadvantaged, in rural areas, and outside the Northern region were most vulnerable to food insecurity, controlling for various confounders. Additionally, low economic status, no occupational pension, currently working, social isolation, physical impairment, functional disabilities, poor self-rated health, and arthritis were associated with an increased risk of food insecurity. Conclusions: More active food assistance programs catering to older adults and a better provision of economic and social security are warranted to establish a food-secure environment for rapidly aging India.

## 1. Introduction

Food insecurity can result in reduced nutritional quality of food eaten by individuals, including both adults and children, leading to poor nutrition, including the lack of intake of essential nutrients such as protein, vitamins, and minerals. The Food and Agriculture Organization (FAO) of the United Nations defines food insecurity as follows: “A person is food insecure when they lack regular access to enough safe and nutritious food for normal growth and development and an active and healthy life” [1]. In the event that access to food is uncertain, individuals may need to sacrifice other basic needs, just to be able to eat. When they are able to access food, they are likely to eat whatever is most readily available or cheapest—such as highly processed foods that are energy-dense and high in saturated fats—resulting in obesity or other forms of malnutrition.

Past studies have shown that food insecurity is associated with a range of adverse health outcomes, including negative nutrition-related outcomes such as having excess weight [2,3] or being underweight [4,5]. In this regard, a systematic review study that examines the association between food insecurity and weight abnormality in adults revealed that the risk of being underweight with severe food insecurity is higher than the risk of being obese [5]. Past studies have also shown that the lack of regular access to enough safe and nutritious food is linked to a range of chronic health conditions, such as cardiovascular diseases [6,7], hypertension [2,8], diabetes [9,10], and physical frailty [11,12,13]. Food insecurity has also been associated with poor mental health and depression [14], sleep disorder [15], poorer subjective well-being [16,17], and poorer cognitive function [18,19].

An international body of literature has also examined the correlates of food insecurity in the United States, Mexico, Australia, and South Korea [20,21,22,23,24,25,26]. Empirical evidence from these studies suggests that there are a variety of factors associated with an increased risk of food insecurity, including functional impairments [20,21,22], chronic diseases and health problems [22,23,24], living alone [23,25], financial vulnerability [21,23,25], and lack of social support [26]. At the same time, the aging process plays a critical role in food and dietary intake. For instance, studies have shown that the body tends to undergo physio-pathological alterations with aging, including alterations of the digestive system, which can compromise an older adult’s capacity to acquire, prepare, and consume foods, contributing to a reduction in dietary intake [27,28]. Older adults may be more susceptible to food insecurity and its adverse health outcomes due to the aging process. Substantial research attention has thus focused on food insecurity and malnutrition among older adults, especially in rapidly aging societies. 

An example of a fast-aging nation is India. The Republic of India is a country in South Asia and is the seventh-largest country by area. India overtook mainland China as the world’s most populous nation in 2023 [29]. In the year 2021, there were 146 million people in India who were 60 years or older. With projected declines in the total fertility rate and increases in life expectancy, this figure is anticipated to increase to 348 million in 2050 and further to 552 million by 2100 [30]. Also worrisome is the fact that India is ranked among the top 20 countries on the 2022 Global Hunger Index, with hunger levels categorized as ‘serious’ [31]. Notably, hunger levels are more severe in India than in countries like Bangladesh, Laos, Nepal, Cambodia, and Ghana. It is estimated that about 6% of Indian adults aged 45 and above have had to reduce the size of their meals, 5% have been hungry due to inadequate food, and 4% have experienced not eating for a whole day because there was not enough food in their household food in the past 12 months [32]. A number of studies focusing on older adults in India have demonstrated that food insecurity is associated with lower levels of cognition [17,18], exhaustion, weight loss, and other aspects of physical frailty [12,13], malnutrition and underweight [4,17], and poor mental health and depressive symptoms [17]. Nonetheless, less is known about the correlates of food insecurity among older adults in India. Given the range of demographic, socio-economic, and health-related factors associated with an increased risk of food insecurity among adults in other countries [20,21,22,23,24,25,26], it is imperative to investigate which factors are most salient for food-insecure older adults in India.

In this paper, we explore the prevalence and correlates of food insecurity in a large population-based sample of adults aged 60 or older in India. Using 2017–2018 data, our study seeks to investigate the factors associated with food insecurity, focusing on the respective roles of sociodemographic, health-related, economic, and social support factors. While health-related factors such as physical impairments and functional disabilities may limit older adults’ access to cooked food or raw food supply, financial difficulties or financial dependency may also contribute to the stress of living with uncertain access to food and anxiety about the household food supply. Understanding the factors associated with food insecurity is thus key to understanding older adults’ specific needs to develop targeted interventions and ultimately decrease the prevalence of food insecurity and improve the quality of life of older adults in rapidly aging, lower-middle-income countries like India. 

## 2. Materials and Methods

### 2.1. Study Design and Sample

The data were from the inaugural wave of the Longitudinal Aging Survey in India (LASI) conducted in 2017–2018. LASI, 2017–2018 is a large-scale nationally representative survey of the health, economic, and social well-being of the Indian population aged 45 and older and their spouses. It covers more than 73,000 older adults from all of the states and union territories in India, and the survey was conducted from April 2017 to December 2018. The LASI used multistage stratified area probability cluster sampling to achieve a nationally representative sample of older adults. The detailed methodology, with complete information on the survey design and data collection, is documented in the survey report [32]. LASI provides comprehensive information on demographics, household economic status, health conditions, family and social networks, work and employment, retirement, satisfaction, and life expectations. The LASI study was approved by the Indian Council of Medical Research Ethics Committee and written/oral informed consent was obtained from the participants [32]. 

**Figure 1:**
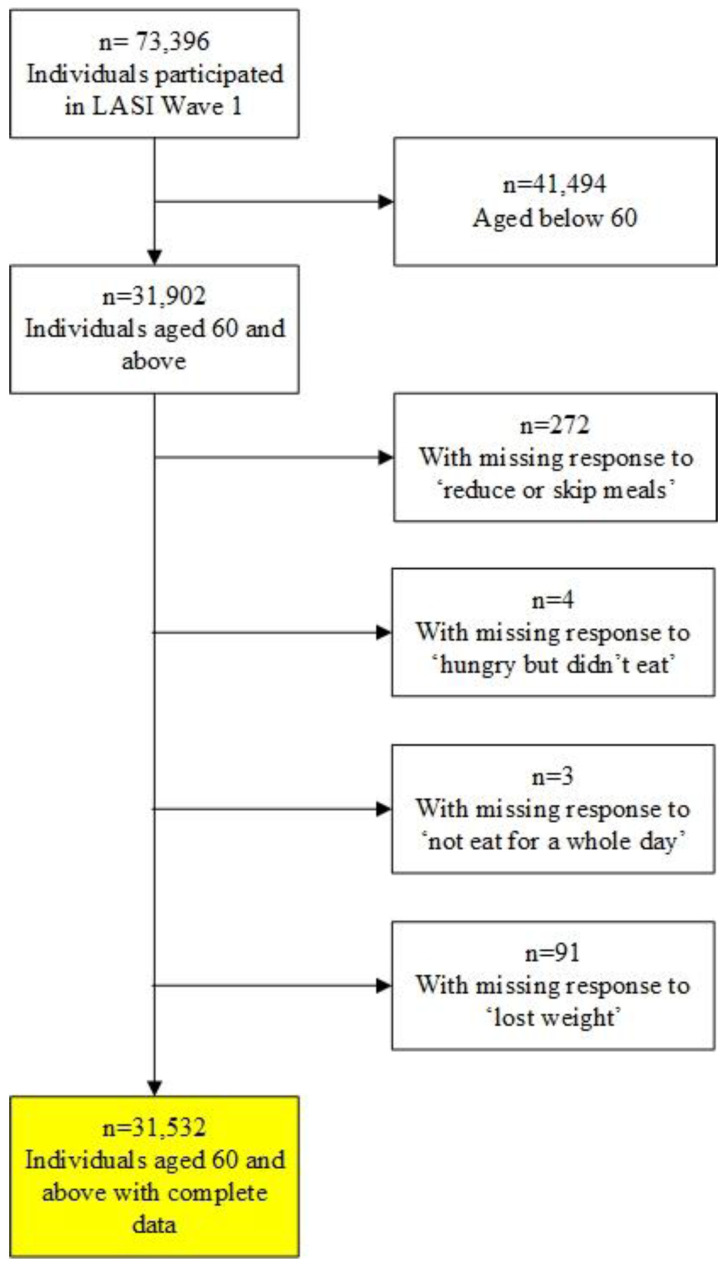
Flowchart of the study sample, LASI, 2017–2018.

The overall response rate for LASI was around 85%. We focused on older adults aged 60 or older. There were 31,902 LASI respondents aged 60 or older, of which 31,532 answered questions on food insecurity (described in the next subsection). Figure 1 provides a flowchart of the sample selection process. Thus, the analytical sample used for the present study was 31,532 older adults aged 60 years and above. 

### 2.2. Measures

#### 2.2.1. Outcome Variable

Food insecurity among older adults was assessed using an adapted version of the Food Insecurity Experience Scale (FIES) that was developed by FAO’s Voices of the Hungry project. Specifically, LASI utilized four items out of the 8-item FIES, which is similar to those items assessed in the Household Food Security Scale, which has been validated in the Indian setting [33,34]. The four food insecurity items in LASI are:

1. In the last 12 months, did you reduce the size of your meals or skip meals because there was not enough food at your household?
2. In the last 12 months, were you hungry but didn’t eat because there was not enough food at your household?
3. In the past 12 months, did you ever not eat for a whole day because there was not enough food at your household?
4. Do you think that you have lost weight in the last 12 months because there was not enough food in your household?

Food insecurity in the current study was defined as an affirmative response to any of these four questions, following past studies [17]. Our outcome variable is an indicator variable set to 1 if food insecure; otherwise, 0. In our sample, the four-item food insecurity measure had a Cronbach’s α reliability coefficient of 0.821, and the pairwise correlation coefficient for items ranged from 0.458 to 0.648. 

#### 2.2.2. Independent Variables

We considered various factors potentially related to food insecurity by drawing from previous studies and grouped the factors as follows. Sociodemographic characteristics included age (60–64, 65–69, 70–74, 75–79, and ≥80 years), sex (1 = female); marital status (1 = married); education attainment (none, less than primary education, primary education, and secondary education or higher); place of residence (1 = rural); and geographical region (North, Central, East, Northeast, West, and South). Following studies based on the Indian context [12,17], we also included caste as a predictor variable. The three categories used were: scheduled caste/scheduled tribe (SC/ST), other backward class (OBC), and others. The SC/STs are generally acknowledged as being the more disadvantaged socio-economic groups in India, while the OBC is the group of people who are identified as educationally, economically, and socially backward. The “others” category comprised those having higher social status [35].

Economic status was measured using the monthly per capita expenditure (MPCE) quintile, which was assessed at the household level. The MPCE is a suitable measure of economic status, especially in relation to the topic of food insecurity, as it is based on questions regarding expenditures on food and non-food items standardized to the 30-day reference period. The MPCE variable was divided into five quintiles (poorest, poorer, middle, richer, and richest). We also controlled for employment status using a binary variable set to 1 if the respondent was currently working. To further examine how economic and financial dependency influences food insecurity, we included three other variables. The first was whether the respondent was currently receiving an occupational pension. An indicator variable set to 1 was constructed for respondents who stated that they were currently receiving a pension from their employers; otherwise, it was 0. The second was whether they received financial help. An indicator variable set to 1 was constructed for respondents who said they had been receiving financial help or support from other household members in the past 12 months; otherwise, 0. The third variable was childhood financial circumstances based on the question: “Now think about your family when you were growing up, from birth to age 16. Compared to other families in your community, would you say your family during that time was pretty well off financially, about average, or poor?” Responses were coded as a categorical variable (pretty well off, average, and poor).

To account for the availability of social support, we included living arrangements, the number of children, and whether the respondents felt alone. The three distinct categories used for living arrangements were living alone, with a spouse, and with others (but not spouse). The number of children alive was treated as a continuous variable. An indicator variable was constructed for social isolation and set to 1 for respondents who stated they felt alone often or most of the time (3 or more days in a week); otherwise, 0. This approach is consistent with the previous literature [36]. Finally, we considered several health-related factors. Physical impairment, which consisted of upper or lower body impairments, was a binary variable. Poor self-rated health was also a binary variable. The inability to perform activities of daily living (ADLs) was used to measure a person’s functional status. Using responses to six ADL questions (including dressing, walking across a room, bathing, eating, getting in or out of bed, or using the toilet), we formed three ADL categories (none, one limitation, two or more limitations). To account for the presence of chronic health conditions, we created seven separate binary variables for hypertension, stroke, chronic heart diseases, cancer, chronic lung disease, diabetes, and arthritis. In sum, the four groups of independent variables used were: sociodemographic, economic, social support, and health-related factors.

### 2.3. Statistical Analysis

Descriptive analysis was performed on the weighted sample, and sample characteristics of the full sample are reported. We also report the sample characteristics of the group of food-secure older adults versus those who were food insecure. Bivariate analysis was conducted to assess intergroup differences based on standard *χ*^2^ tests (one-sided) for categorical data and *t* tests (two-sided) for continuous data. Given that India is a large country and the prevalence of food insecurity may vary across states and territories, we present a descriptive analysis of the prevalence of food insecurity by geographical region (North, Central, East, Northeast, West, and South). Hierarchical logistic regressions were then implemented to evaluate the risk factors associated with food insecurity for the study sample. Model 1 included only the sociodemographic factors. Model 2 added the economic factors, while the social support factors were added in Model 3. Model 4 contained all the above-mentioned independent variables. To avoid overfitting the model and address possible issues of collinearity, we estimated the variance inflation factor to check the multi-collinearity among the independent variables; however, it was found that there was no evidence of multi-collinearity among the variables. The regression estimates are presented in the form of adjusted odds ratios (ORs) with a 95% confidence interval (CI). Individual-level weights from the LASI data were used to make the estimates nationally representative. All statistical analyses were carried out using STATA version 16.0 (StataCorp, College Station, TX, USA).

## 3. Results

### 3.1. General Characteristics of the Study Population

Table 1 presents the characteristics of the participants in the study sample (*N* = 31,532). In the weighted sample, women accounted for nearly 53% and men for approximately 47% of the sample. About two-fifths of the respondents were below 70 years old, with the rest in more advanced age groups. The study sample consisted of about 62% who were married, 71% who resided in rural areas, and 31% who were currently working. About 57% of the respondents had no education, while the rest had some education. Only 6% and 15% of the older adults received occupational pension and financial help, respectively. Most respondents had poor or average childhood financial circumstances. About 61% lived with spouse, 33% with others, and 6% lived alone. About 15% reported they felt alone often or most of the time. The majority of the community-dwelling respondents (76%) had no ADL limitations, and the prevalence of chronic conditions ranged from 0.7% to 33%. Only 6% reported a physical impairment. 

### 3.2. Prevalence of Food Insecurity and Variation by Region

The overall prevalence of food insecurity was about 10.5% in the weighted sample. Comparing the sample characteristics for respondents who were food-secure versus those who were food insecure in Table 1, we found that lower proportions of those who were married, educated, resided in urban areas, and had no caste/tribe were food insecure compared to the total sample. Older Indian adults with food insecurity issues were also more likely to be currently working, not receiving an occupational pension, of lower economic status, financially poor in childhood, living alone, and socially isolated (*p* < 0.001 in all cases). Finally, food-insecure respondents were more likely to have physical impairments, ADL limitations, and arthritis, although diabetes and hypertension were somehow less prevalent among this group.

**Figure 2:**
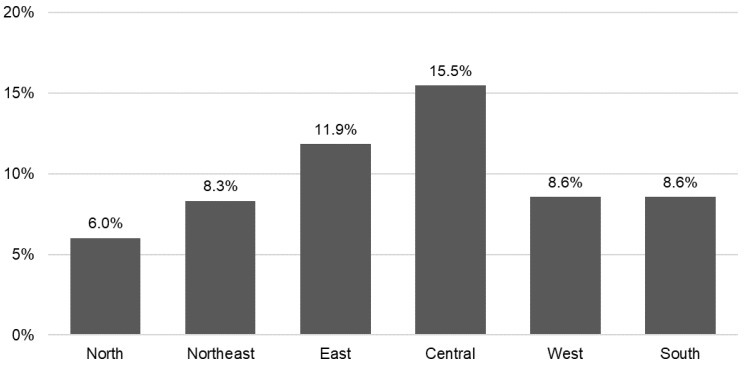
Prevalence rate of food insecurity by region in India, LASI, 2017–2018. Notes: *N* = 31,532; individual-level weights are applied.

The observed prevalence rate of food insecurity (10.5%) in our study sample is consistent with that reported in previous studies using the LASI dataset [17]. Nonetheless, India is a geographically large country. It is possible that the prevalence of food insecurity may vary across states and territories. Figure 2 provides some insights into the variation in the prevalence rate of food insecurity across sub-national regions (North, Central, East, Northeast, West, and South). We see that larger proportions of older adults in the Eastern and Central regions were food insecure compared to their counterparts elsewhere. The food insecurity prevalence rate was highest in the Central region at 16%, followed by the East region at 12%. This was attributable to poorer states like Madhya Pradesh, Bihar, and Jharkhand in these two regions, where many people live below the poverty line. The average prevalence in the other regions was around 6–9%. In our weighted sample, about 45% of the study participants resided in the Eastern and Central regions, 16% in the Northern regions, 17% in the West, and 22% in the South. 

### 3.3. Multivariate Regression Results

Table 2 shows the hierarchical logistic regression results. The adjusted odds ratios (ORs) and the accompanying 95% confidence intervals are reported. We found that all the sociodemographic factors (age, sex, marital status, education, caste, residence, and region) were important explanatory factors for food insecurity among older adults. The estimates in Model 1 show that the odds of being food insecure were significantly lower for females, married persons, those aged 80 or older, those who completed primary education, those less socially disadvantaged, those residing in urban areas, and those in the Northern regions of India. These relationships continued to hold even when more covariates were entered into the model, although the magnitudes of some associations were attenuated somewhat. For example, older adults in rural areas were 1.64 times more likely to be food insecure than their counterparts in urban areas in Model 1. In the full model (Model 4), the adjusted odds ratio was slightly lower at 1.53 (95% CI 1.31–1.78, *p* < 0.001). Also, the full model shows that older adults living outside the Northern region, and particularly those in the Central and East regions, faced increased risk of food insecurity as compared to those living in the North. These adjusted estimates are consistent with our earlier results from Figure 2.

Among the economic factors included in Model 2, the MPCE quintile and receiving an occupational pension were predictive of food insecurity. The final model confirms that both factors were inversely associated with food insecurity in the presence of other covariates. Persons in the middle MPCE quintile were less likely to be food insecure than the poorest (OR 0.77, 0.65–0.91, *p* < 0.01), as were those who currently received an occupational pension (OR 0.62, 0.43–0.88, *p* < 0.01). In addition, Model 4 reveals that currently working older adults were at higher risk of food insecurity. Of the three social support factors added in Model 3, only social isolation was significantly associated with the outcome. Older adults who felt alone often or most of the time were twice as likely to be food insecure than those who did not (OR 2.09, 1.80–2.43, *p* < 0.001). Living arrangements and the number of children were not associated with food insecurity. In terms of health-related factors, having a physical impairment (OR 1.68, 1.34–2.11), having two or more ADL limitations (OR 1.72, 1.41–2.08), and poor self-rated health (OR 1.47, 1.27–1.69) were positively and strongly associated with higher odds of food insecurity (*p* < 0.001 in all cases). Weaker associations at *p* < 0.05 were observed between certain chronic conditions and food insecurity: older adults with arthritis were more likely to be food insecure (OR 1.18, 1.01–1.38), while diabetics were less likely (OR 0.79, 0.65–0.97). The *R*-squared statistic increased from 0.042 to 0.086 moving from Model 1 to Model 4, suggesting that the model fit improved as the economic, social, and health factors were sequentially added.

## 4. Discussion

Given the globally increasing relevance of food insecurity, the present study examined the prevalence of food insecurity and the risk factors associated with household food insecurity among older adults in India. The prevalence of food insecurity was 10.5% in the nationally representative study population of older adults, and this varied somewhat by geographical region. Our multivariate analysis shows that all the sociodemographic factors (age, sex, marital status, education, caste, residence, and region) were important in explaining food insecurity among older adults, even after controlling for various confounders. This is notable because the majority of these factors are immutable from a policy perspective. Older adults who were male, residing in rural areas, with lower educational attainment, and socially disadvantaged were most vulnerable to food insecurity. These findings can be rationalized in part by the fact that rural households in India, as well as those belonging to a caste/tribe, are mostly clustered in isolated areas with limited market access [37], which hinders accessibility to and availability of food and food consumption. 

Another finding from this study is the association between food insecurity and geographical region. Controlling the other covariates, we found that respondents not living in the Northern region faced increased risk of food insecurity compared to the reference group. Older adults living in the Central and East regions were especially vulnerable, and were 2.43 and 1.63 times more likely, respectively, to be food insecure compared to those living in the North. This result is consistent with the known regional disparities in wealth and other socio-economic characteristics across states in India. In particular, the Central and East regions of India (comprising states like Bihar, Jharkhand, Madhya Pradesh, Odisha, and Uttar Pradesh) tend to perform more poorly in key economic indicators, such as the poverty headcount ratio, unemployment rate, and School Education Quality Index. Notably, six out of the seven lowest-income states in India are located in the Central and Eastern regions [38].

As in previous studies conducted elsewhere [20,21,22,23,24], physical impairment, functional disabilities, and chronic diseases were positively associated with food insecurity. Upper or lower body impairments may indeed contribute to food insecurity since older adults with restricted mobility due to physical impairments may face serious challenges accessing food outside or inside their homes. The presence of functional disabilities had a similar effect; the odds ratio estimates for ADL disabilities were approximately of similar magnitude as physical impairment. Older Indian adults with one ADL disability were 1.53 times more likely to be food insecure—and those with multiple ADL disabilities were 1.72 times more likely to be food insecure—than their able counterparts. The prevalence of chronic diseases alongside inadequate health care access is widely acknowledged as a main cause of food insecurity. Our study, however, shows that not all types of chronic diseases are contributing factors. Among the seven chronic conditions considered, only two were significantly associated with food insecurity (albeit only at the 5% level). Having arthritis increased the risk of being food insecure, whereas the presence of diabetes decreased the risk. 

Other than health and sociodemographic characteristics, this study establishes that economic and social support factors also play a critical in explaining food security among the older adult population. Higher economic status was a protective factor against food insecurity, with older adults in the higher MPCE quintiles facing significantly lower odds of food insecurity than those in the poorest quintile. Respondents who were receiving work-related pension income also had lower odds of food insecurity, even though it should be noted that these were retirees who had exited the workforce. In contrast, older adults who were currently still working had higher odds of food insecurity. While this might seem surprising at first glance, it could be that having to work proxied for those persons who were less financially stable and so understandably had greater difficulties affording healthy and nutritious foods. Nonetheless, the relationship between employment status and food insecurity should be further studied, especially in settings such as India, where there is a large informal workforce comprising workers with no written contract, paid leave, or other benefits.

Our finding that socially isolated older Indian adults were significantly more susceptible to food insecurity is consistent with past studies that have documented a strong positive relationship between loneliness and food insecurity [25,26,39]. Older adults living in rural or remote areas are particularly vulnerable to loneliness because towns and houses are further apart and they may have greater difficulty finding someone to assist them with accessing food, especially if they are homebound and cannot get to a supermarket or grocery store themselves. Also, limited social networks and reduced social capital may mean that older persons have more difficulty accessing food and accessing programs that would potentially help them to become more food secure. Being socially isolated also may mean that older adults rely on an unbalanced diet of convenient processed foods (e.g., frozen foods) or skip meals due to lack of socialization.

The strengths of this present study are its community-based, prospective design, and a nationally representative sample of older adults in India. Nonetheless, there are some limitations. First, it is based on cross-sectional data, which did not include information about the duration or pattern of food insecurity. This limits the ability to study how the factors may correlate with the frequency and duration of food insecurity. Also, because of the cross-sectional nature of the study, a causal association among the factors and food insecurity cannot be estimated. It is possible that reverse causality may be an issue for some of the factors considered, such as diabetes. For example, food-insecure persons may be more likely to consume highly processed foods resulting in a greater likelihood of having diabetes. Future research with richer data on health conditions, nutrition, and food security is required to more closely investigate the causal pathways between food insecurity and certain health conditions. Second, the food insecurity measure and several other factors used were self-reported. These variables might be subject to recall bias or imprecise measurement, and some variables, such as receipt of financial help and poor childhood financial circumstances, might be underestimated. 

## 5. Conclusions

Food insecurity is a pressing public health issue worldwide, especially in middle- and low-income countries, such as India. Not only is India ranked among the top 20 countries on the 2022 Global Hunger Index, but it is also aging rapidly. Our study focuses on the prevalence and risk factors of food insecurity in India’s growing older population, which is distinct from other age groups. The factors that were significantly associated with an increased risk of food insecurity included low economic status, no occupational pension, currently working, social isolation, physical impairment, ADL disabilities, poor self-rated health, and arthritis. Also, older adults who were male, younger, lowly-educated, socially disadvantaged, in rural areas, and living outside the Northern region were most vulnerable to food insecurity. These findings will be useful for identifying segments of the older population to be targeted by interventions. In addition, active food assistance programs cater to older persons living below the poverty line, and a better provision of economic and social security is needed in order to establish a food-secure environment for the rapidly aging society of India.

## Figures and Tables

**Table 1:** Comparison of respondents with and without food insecurity.

Characteristic	Total	Food Insecure		*p*-Value
		No	Yes	
*Number of respondents*	*31,532*	*28,229*	*3303*	
*(in %)*	*(100%)*	*(89.5%)*	*(10.5%)*	
Age groups				0.657
60–64	30.0%	29.8%	31.7%	
65–69	28.8%	28.9%	27.4%	
70–74	18.8%	18.8%	18.7%	
75–79	11.2%	11.1%	11.4%	
80 and above	11.3%	11.4%	10.7%	
Sex				0.741
Male	47.2%	47.2%	46.7%	
Female	52.8%	52.8%	53.3%	
Marital status				<0.001
Married/cohabiting	61.9%	62.5%	56.7%	
Others	38.1%	37.5%	43.3%	
Education				<0.001
None	56.8%	55.3%	68.9%	
Less than primary	11.5%	11.4%	12.7%	
Primary	11.2%	11.6%	8.1%	
Secondary or higher	20.5%	21.7%	10.3%	
Caste				<0.001
SC/ST	27.3%	26.2%	36.0%	
OBC	45.3%	45.6%	43.3%	
No caste/tribe	27.4%	28.2%	20.7%	
Place of residence				<0.001
Urban	29.0%	30.5%	16.3%	
Rural	71.0%	69.5%	83.7%	
Region				<0.001
North	12.7%	13.3%	7.3%	
Northeast	3.0%	3.1%	2.4%	
East	23.7%	23.3%	26.8%	
Central	21.2%	20.0%	31.3%	
West	17.1%	17.5%	14.0%	
South	22.4%	22.9%	18.3%	
Currently working				<0.001
No	69.2%	69.7%	64.6%	
Yes	30.8%	30.3%	35.4%	
MPCE quintile				<0.001
Poorest	21.8%	21.0%	27.8%	
Poorer	21.8%	21.6%	23.1%	
Middle	20.7%	20.9%	18.3%	
Richer	19.3%	19.6%	17.1%	
Richest	16.5%	16.8%	13.7%	
Receive occupational pension				<0.001
No	94.3%	93.9%	97.9%	
Yes	5.7%	6.1%	2.1%	
Receive financial help				0.047
No	85.0%	85.2%	83.2%	
Yes	15.0%	14.8%	16.8%	
Childhood SES				<0.001
Pretty well off	8.4%	8.7%	6.2%	
Average	48.8%	50.0%	39.1%	
Poor	42.7%	41.4%	54.7%	
Living arrangement				<0.001
With spouse	60.8%	61.4%	56.0%	
With others (but not spouse)	33.5%	33.7%	32.3%	
Live alone	5.7%	5.0%	11.7%	0.673
Number of children	3.7	3.7	3.8	
(SD)	(2.0)	(1.9)	(2.1)	
Social isolation				<0.001
Not isolated	85.5%	86.9%	73.4%	
Isolated	14.5%	13.1%	26.6%	
Physical impairment				<0.001
No	94.1%	94.7%	89.6%	
Yes	5.9%	5.3%	10.4%	
ADL limitation				<0.001
No	76.3%	77.6%	65.0%	
One limitation	9.6%	9.1%	13.9%	
2 or more limitation	14.2%	13.4%	21.1%	
Poor self-rated health				<0.001
No	76.3%	77.6%	65.8%	
Yes	23.7%	22.4%	34.2%	
Angina/heart disease				0.070
No	94.8%	94.7%	96.0%	
Yes	5.2%	5.3%	4.0%	
Arthritis				0.021
No	80.4%	80.7%	77.6%	
Yes	19.6%	19.3%	22.4%	
Chronic lung disease				0.156
No	91.5%	91.7%	90.2%	
Yes	8.5%	8.3%	9.8%	
Diabetes				<0.001
No	85.8%	85.1%	90.9%	
Yes	14.2%	14.9%	9.1%	
Hypertension				0.006
No	67.2%	66.8%	70.8%	
Yes	32.8%	33.2%	29.2%	
Stroke				0.063
No	97.3%	97.4%	96.6%	
Yes	2.7%	2.6%	3.4%	
Cancer				0.483
No	99.3%	99.3%	99.4%	
Yes	0.7%	0.7%	0.6%	

Notes: SC/ST = scheduled tribe/caste; OBC = other backward class; MPCE = monthly per capita expenditure; SES = socio-economic status; SD = standard deviation; ADL = activity of daily living. For categorical variables, the numbers shown are percentages. For number of children, the mean values are presented, and the standard deviations are shown in parentheses. The *p*-values are calculated using the *t*-test and the chi-squared test for continuous and categorical variables, respectively. Individual-level weights are applied.

**Table 2:** Factors associated with food insecurity (hierarchical logistic regressions).

	OR (95% CI)			
Variable	Model 1	Model 2	Model 3	Model 4
Age groups: 60–64 (ref.)				
65–69	0.86	0.87	0.88	0.86
	(0.73–1.00)	(0.74–1.02)	(0.75–1.03)	(0.73–1.00)
70–74	0.85	0.88	0.89	0.85
	(0.70–1.02)	(0.73–1.06)	(0.74–1.08)	(0.70–1.03)
75–79	0.84	0.87	0.88	0.79 *
	(0.67–1.05)	(0.69–1.10)	(0.70–1.11)	(0.63–0.99)
80 and above	0.71 **	0.74 *	0.77 *	0.65 ***
	(0.56–0.89)	(0.58–0.95)	(0.60–0.98)	(0.50–0.83)
Female	0.80 **	0.82 *	0.80 **	0.80 **
	(0.69–0.93)	(0.70–0.96)	(0.69–0.94)	(0.69–0.94)
Married/cohabiting	0.74 ***	0.74 ***	0.60	0.59 *
	(0.64–0.86)	(0.64–0.86)	(0.35–1.02)	(0.35–0.99)
Education: None (ref.)				
Less than primary	0.96	0.98	0.97	0.96
	(0.79–1.17)	(0.80–1.19)	(0.80–1.19)	(0.79–1.18)
Primary	0.63 ***	0.67 ***	0.68 **	0.70 **
	(0.50–0.80)	(0.53–0.85)	(0.54–0.86)	(0.55–0.87)
Secondary or higher	0.46 ***	0.55 ***	0.54 ***	0.58 ***
	(0.37–0.58)	(0.44–0.70)	(0.43–0.68)	(0.47–0.73)
Caste: SC/ST (ref.)				
OBC	0.80 **	0.83 *	0.83 *	0.83 *
	(0.69–0.92)	(0.71–0.96)	(0.71–0.96)	(0.72–0.97)
No caste/tribe	0.75 ***	0.80 *	0.82 *	0.82 *
	(0.63–0.89)	(0.68–0.96)	(0.69–0.98)	(0.69–0.98)
Rural	1.64 ***	1.63 ***	1.58 ***	1.53 ***
	(1.42–1.91)	(1.40–1.89)	(1.35–1.84)	(1.31–1.78)
Region: North (ref.)				
Northeast	1.36 *	1.38 *	1.47 **	1.45 **
	(1.06–1.75)	(1.07–1.78)	(1.14–1.90)	(1.12–1.87)
East	1.98 ***	1.83 ***	1.84 ***	1.63 ***
	(1.66–2.37)	(1.53–2.20)	(1.53–2.20)	(1.35–1.97)
Central	2.73 ***	2.60 ***	2.54 ***	2.43 ***
	(2.24–3.32)	(2.12–3.18)	(2.07–3.10)	(1.98–2.98)
West	1.62 ***	1.47 **	1.48 **	1.33 *
	(1.29–2.04)	(1.17–1.86)	(1.17–1.88)	(1.05–1.69)
South	1.58 ***	1.66 ***	1.47 ***	1.25 *
	(1.30–1.93)	(1.36–2.03)	(1.20–1.81)	(1.02–1.54)
Currently working		1.07	1.06	1.21*
		(0.92–1.25)	(0.91–1.24)	(1.04–1.42)
MPCE quintile: Poorest (ref.)				
Poorer		0.86	0.89	0.89
		(0.72–1.02)	(0.75–1.07)	(0.75–1.07)
Middle		0.77 **	0.77 **	0.77 **
		(0.64–0.91)	(0.65–0.92)	(0.65–0.92)
Richer		0.82	0.82	0.81 *
		(0.67–1.01)	(0.67–1.00)	(0.66–1.00)
Richest		0.87	0.84	0.82
		(0.71–1.07)	(0.68–1.03)	(0.67–1.02)
Receiving occupational pension		0.59 **	0.59 **	0.62 **
		(0.42–0.84)	(0.42–0.84)	(0.43–0.88)
Receiving financial help		1.11	1.03	1.01
		(0.95–1.29)	(0.88–1.21)	(0.86–1.18)
Childhood SES: Pretty well off (ref.)				
Average		0.91	0.88	0.92
		(0.70–1.19)	(0.68–1.15)	(0.71–1.19)
Poor		1.23	1.20	1.20
		(0.94–1.62)	(0.92–1.58)	(0.92–1.57)
Living arrangement: With spouse (ref.)				
With others (but not spouse)			0.62	0.61
			(0.37–1.06)	(0.37–1.04)
Living alone			1.26	1.25
			(0.71–2.24)	(0.71–2.20)
Number of children			0.99	0.99
			(0.95–1.02)	(0.95–1.02)
Socially isolated			2.27 ***	2.09 ***
			(1.95–2.63)	(1.80–2.43)
Has physical impairment				1.68 ***
				(1.34–2.11)
ADL limitation: No (ref.)				
One limitation				1.53 ***
				(1.29–1.81)
2 or more limitations				1.72 ***
				(1.41–2.08)
Poor self-rated health				1.47 ***
				(1.27–1.69)
Angina/heart disease				0.97
				(0.71–1.32)
Arthritis				1.18 *
				(1.01–1.38)
Chronic lung disease				1.14
				(0.90–1.44)
Diabetes				0.79 *
				(0.65–0.97)
Hypertension				1.07
				(0.93–1.24)
Stroke				1.02
				(0.73–1.43)
Cancer				1.06
				(0.57–2.00)
N (unweighted)	31,532	31,532	31,532	31,532
R-squared	0.042	0.047	0.068	0.086
AIC	2396.08	2383.19	2331.00	2286.85

Notes: SC/ST = scheduled tribe/caste; OBC = other backward class; MPCE = monthly per capita expenditure; SES = socio-economic status; ADL = activity of daily living; AIC = Akaike information criterion. Odds ratios from the logistic regressions are reported, together with the 95% CIs in parentheses. Individual-level weights are used in the analysis; see text. *** *p* < 0.001, ** *p* < 0.01, * *p* < 0.05.

## Data Availability

The data are publicly available for download to registered users on the LASI website (https://lasi-india.org/ (accessed on 1 June 2023)).
